# Impact of Myeloproliferative neoplasms on patients’ employment status and work productivity in the United States: results from the living with MPNs survey

**DOI:** 10.1186/s12885-018-4322-9

**Published:** 2018-04-13

**Authors:** Jingbo Yu, Shreekant Parasuraman, Dilan Paranagama, Andrew Bai, Ahmad Naim, David Dubinski, Ruben Mesa

**Affiliations:** 1 Incyte Corporation, 1801 Augustine Cut-Off, Wilmington, DE 19803 USA; 2 UT Health San Antonio Cancer Center, San Antonio, TX USA

**Keywords:** Employment, Essential thrombocythemia, Myelofibrosis, Myeloproliferative neoplasm, Polycythemia vera, Work productivity

## Abstract

**Background:**

Patients with the myeloproliferative neoplasms (MPNs) myelofibrosis (MF), polycythemia vera (PV), and essential thrombocythemia (ET) are at increased risk for thrombotic and cardiovascular events and experience a variety of burdensome symptoms. However, there is a paucity of data in the biomedical literature about how MPNs impact productivity in the workplace. This analysis of the Living with MPNs survey was conducted to evaluate the impact of MPNs on employment, career potential, and work productivity.

**Methods:**

This cross-sectional online survey included respondents aged 18–70 years living in the United States with a diagnosis of MF, PV, or ET. The survey consisted of ~ 100 questions related to MPN diagnosis, disease-related medical history, MPN-related symptoms and functional status, changes in employment and work productivity, and impact on daily activities since diagnosis. The MPN Symptom Assessment Form Total Symptom Score (MPN-SAF TSS) was used to assess symptom burden. The Work Productivity and Activity Impairment Specific Health Problem questionnaire (WPAI-SHP) was used to assess the effects of MPNs on work productivity and activity (7-day recall) among currently employed respondents. Correlations between MPN-SAF TSS and WPAI-SHP scores were calculated using Spearman’s coefficients.

**Results:**

Of 904 respondents, 592 were employed (MF, *n* = 174; PV, *n* = 248; ET, *n* = 170) at the time of their MPN diagnosis. Approximately half (50.5%) of the 592 employed survey respondents reported ≥1 change in employment status because of their diagnosis, most commonly “left a job” (30.2%) “went on medical disability leave” (24.8%), and “had reductions in work hours for at least 3 months” (21.8%). Among respondents who remained employed at the time of survey participation (*n* = 398), mean WPAI-SHP scores were as follows: absenteeism, 6.9%; presenteeism, 27.4%; overall work impairment, 31.1%; and activity impairment, 32.8%. WPAI-SHP scores positively correlated with MPN-SAF TSS (correlation coefficients, 0.37–0.70; *P* < 0.001).

**Conclusions:**

Half of the employed respondents had an employment status change (eg, leaving a job, medical disability leave, early retirement) because of their disease since the diagnosis. Currently employed respondents reported meaningful impairments in work productivity and activities of daily living that were attributable to their MPNs, and the degree of impairments highlighted the severity of symptom burden.

**Electronic supplementary material:**

The online version of this article (10.1186/s12885-018-4322-9) contains supplementary material, which is available to authorized users.

## Background

The Philadelphia-negative myeloproliferative neoplasms (MPNs), myelofibrosis (MF), polycythemia vera (PV), and essential thrombocythemia (ET) [1], are chronic disorders associated with burdensome signs and symptoms [2, 3] and increased risk of mortality compared with the age- and sex-matched general population (10-year relative survival rates: MF, 0.21; PV, 0.64; ET, 0.68) [4]. In the United States, the prevalence of MPNs has been estimated to be 88 to 120 per 100,000 residents (MF, 4–6; PV, 45–57; ET, 39–57) [5].

Patients with MPNs experience a pronounced symptom burden that may include fatigue, night sweats, bone pain, itching, concentration problems, and splenomegaly-related symptoms, which compromise activities of daily living and quality of life [2, 3]. The MPN Landmark survey evaluated the patient-reported symptom burden of MF, PV, and ET among patients in the United States [3]. Regardless of MPN subtype, fatigue was the most severe and common symptom (MF, 80%; PV, 73%; ET, 71%). Abdominal discomfort and night sweats were reported by approximately half of the patients with MF (53% and 51%, respectively). Itching and night sweats were reported by 55% and 45% of patients with PV, respectively. Over a third of patients with ET reported night sweats and concentration problems (38% and 35%, respectively) [3].

Studies have shown that chronic diseases are associated with negative economic impacts, not only because of direct medical costs, but also because patient contributions to the work force are reduced or eliminated [6, 7]. Cancer diagnoses have been associated with reductions in household income [8]. In addition, chronic diseases have negative macroeconomic effects [6].

Few studies have explored the impact of MPNs on employment status, career potential, and work productivity. The MPN Landmark survey indicated that many employed patients experienced a change in employment such as reduction in work hours, need for sick days, and job termination or early retirement [3]. This analysis from the Living With MPNs survey aimed to further assess the impact of MPNs on employment status, career potential, and work productivity of patients in the United States.

## Methods

### Study design and respondents

The Living with MPNs survey was a cross-sectional survey study conducted in the United States. Respondents who were between 18 and 70 years of age living in the United States with a diagnosis of MF, PV, or ET were eligible to participate in the survey. The survey was administered online between April and November of 2016 [9]. Respondents who consented to participate and completed the survey were offered an optional $25 compensation.

Respondents were recruited to the survey through posts on MPN-focused social media (eg, patient group Facebook pages), emails and/or post cards sent by MPN groups, posts on patient advocacy websites, banner ads at selected medical websites, text or banner ads through Google or Facebook, and postcards sent to hematologist/oncologist offices for distribution. Data capture was performed online using an electronic form in which answers to all eligible survey questions were mandatory.

### Survey instrument

The survey questionnaire included approximately 100 MPN-related questions, although the survey length for individual respondents varied depending on responses. The questions focused on MPN diagnosis, disease-related history, MPN-related symptoms, functional status, changes in employment status since diagnosis, and changes in work productivity and daily activities. For respondents who indicated that they were employed full- or part-time by an employer (ie, not self-employed) at the time of MPN diagnosis, a subset of questions related to employment (change of employment, time, and related income) was administered (Additional file 1: Table S1); this analysis includes descriptive results of data collected using these questions.

Effects of MPN diseases on work productivity and activity for currently employed respondents in the preceding 7 days were assessed using the Work Productivity and Activity Impairment Specific Health Problem questionnaire (WPAI-SHP version 2.0) [10, 11]. The WPAI-SHP evaluated absenteeism (percentage of work time missed in the past 7 days because of an MPN), presenteeism (percentage of impairment experienced while at work in the past 7 days because of an MPN), overall work impairment (percentage of work time missed or impaired in the past 7 days because of either absenteeism or presenteeism), and activity impairment (percentage of impairment in daily activities in the past 7 days because of an MPN).

The MPN Symptom Assessment Form Total Symptom Score (MPN-SAF TSS) is a 10-item questionnaire designed to assess MPN symptom burden [2]. The data reported include descriptive results related to fatigue, concentration, early satiety, inactivity, night sweats, itching, bone pain, abdominal discomfort, weight loss, and fever. Spearman coefficients were calculated to evaluate correlations between MPN-SAF TSS and WPAI-SHP scores.

## Results

### Demographics of respondents

The survey was completed by 904 respondents with MPNs (MF, *n* = 270; PV, *n* = 393; ET, *n* = 241). At the time of MPN diagnosis, 65.5% (*n* = 592) of respondents were employed full (53.2%) or part time (12.3%) by an employer (ie, not self-employed), 9.6% were retired, 7.2% were self-employed, 6.5% were disabled, 6.4% were homemakers, and 4.8% were unemployed.

Among employee respondents at time of diagnosis, mean age among respondents who were employed at diagnosis was 54.0 years (Table 1). The majority of respondents were women (70.6%) and were married or had domestic partners (72.3%). Half of all respondents completed a bachelor’s degree or higher (50.0%; Table 1). On average, respondents had been diagnosed with an MPN for 6.1 years at the time of the survey (Table 2). Of the comorbid conditions that were assessed in the survey, the most commonly reported were diabetes (9.8%), solid tumor (5.7%), and emphysema or chronic obstructive pulmonary disease (4.6%). Overall, 21.6% of respondents reported a history of thrombotic events.

**Table 1:** Demographics and Employment Status for Respondents With MPNs Employed at Diagnosis

	MF (*n* = 174)	PV (*n* = 248)	ET (*n* = 170)	All MPNs (*n* = 592)
Age, mean (SD), y	57.5 (9.8)	54.5 (9.1)	49.6 (12.2)	54.0 (10.7)
Sex, n (%)				
Female	106 (60.9)	173 (69.8)	139 (81.8)	418 (70.6)
Male	68 (39.1)	75 (30.2)	31 (18.2)	174 (29.4)
Highest level of education, n (%)				
High school completion or less	29 (16.7)	37 (14.9)	10 (5.9)	76 (12.8)
Some college/associate degree	65 (37.4)	90 (36.3)	65 (38.2)	220 (37.2)
Bachelor’s degree	46 (26.4)	64 (25.8)	60 (35.3)	170 (28.7)
Advanced degree	34 (19.5)	57 (23.0)	35 (20.6)	126 (21.3)
Marital status, n (%)*				
Married/domestic partnership	118 (67.8)	188 (76.1)	121 (71.2)	427 (72.3)
Divorced	35 (20.1)	31 (12.6)	25 (14.7)	91 (15.4)
Single (never married)	8 (4.6)	20 (8.1)	17 (10.0)	45 (7.6)
Widowed	11 (6.3)	4 (1.6)	4 (2.4)	19 (3.2)
Separated	2 (1.1)	4 (1.6)	3 (1.8)	9 (1.5)
Employment status at MPN diagnosis, n (%)				
Full-time (≥40 h/wk)	139 (79.9)	203 (81.9)	139 (81.8)	481 (81.3)
Part-time (< 40 h/wk)	35 (20.1)	45 (18.1)	31 (18.2)	111 (18.8)

ET, essential thrombocythemia; MF, myelofibrosis; MPN, myeloproliferative neoplasm; PV, polycythemia vera

*One respondent with PV did not provide information; percentages are based on *n* = 247 (PV) and *n* = 591 (all MPNs)

**Table 2:** Disease Characteristics for Respondents With MPNs Employed at Diagnosis

	MF (*n* = 174)	PV (*n* = 248)	ET (*n* = 170)	All MPNs (*n* = 592)
Duration of disease, mean (SD), y	4.6 (4.7)	6.9 (7.2)	6.3 (6.4)	6.1 (6.4)
Risk score at any time during treatment				
High	25 (14.4)	35 (14.1)	41 (24.1)	101 (17.1)
Intermediate	61 (35.1)	29 (11.7)	21 (12.4)	111 (18.8)
Low	17 (9.8)	22 (8.9)	34 (20.0)	73 (12.3)
Not available or did not recall	71 (40.8)	162 (65.3)	74 (43.5)	307 (51.9)
Comorbid conditions ever diagnosed*				
Diabetes	28 (16.1)	21 (8.5)	9 (5.3)	58 (9.8)
Solid tumor	13 (7.5)	12 (4.8)	9 (5.3)	34 (5.7)
Emphysema or COPD	9 (5.2)	13 (5.2)	5 (2.9)	27 (4.6)
Connective tissue disorders	8 (4.6)	8 (3.2)	10 (5.9)	26 (4.4)
Moderate to severe kidney disease	13 (7.5)	8 (3.2)	3 (1.8)	24 (4.1)
Liver disease	7 (4.0)	9 (3.6)	4 (2.4)	20 (3.4)
History of thrombotic events,^†^ n (%)	40 (23.0)	51 (20.6)	37 (21.8)	128 (21.6)

COPD, chronic obstructive pulmonary disease; ET, essential thrombocythemia; MF, myelofibrosis; MPN, myeloproliferative neoplasm; PV, polycythemia vera

*Occurring in > 3% of all respondents employed at diagnosis

^†^Each respondent could have > 1 thrombotic event

### Impact of Myeloproliferative neoplasms on employment status

**Fig. 1:**
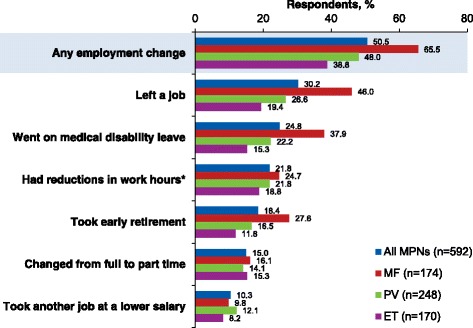
Employment change due to MPNs since diagnosis. ET, essential thrombocythemia; MF, myelofibrosis; MPN, myeloproliferative neoplasm; PV, polycythemia vera. *For ≥3 months

Approximately half (50.5%) of all respondents who were employed at diagnosis (MF, 65.5%; PV, 48.0%; ET, 38.8%) reported making at least 1 change in employment status as a result of their MPN (Figure 1). The most commonly reported MPN-related effects on employment status were “left a job,” “went on medical disability leave,” and “had reductions in work hours for at least 3 months.” When considering the first change in employment status after diagnosis, in the MF and PV cohorts, the most commonly reported response was “left a job” (48.3% and 37.8%, respectively). In the ET cohort, the most common first change was “reducing work hours for at least 3 months” (33.3%). The first change in employment status occurred 2.1 years on average after MPN diagnosis (MF, 1.4 years; PV, 2.9 years; ET, 1.9 years).

Among respondents who “left a job” (MF, *n* = 80; PV, *n* = 66; ET, *n* = 33), the average time from diagnosis to the first time leaving a job was 1.9, 3.9, and 3.3 years, respectively. Of these, most remained unemployed until the time of the survey (MF, 90.0%; PV, 68.2%; ET, 63.6%). Among respondents who returned to work, approximately two-thirds reported a decreased salary compared with the job they left (MF, 62.5%; PV, 61.9%; ET, 58.3%).

For respondents who reported taking early retirement, the average time from diagnosis to retirement was 2.4 years for patients with MF, 4.5 years for patients with PV, and 4.0 years for patients with ET. On average, these respondents retired 7.7, 9.7, and 7.8 years earlier than planned, respectively.

Among patients who took medical disability leave, the average time from diagnosis to first time taking disability leave was 2.2 years (MF), 2.6 years (PV), and 1.8 years (ET). Some respondents reported taking medical disability leave more than once (MF, 19.7%; PV, 32.7%; ET, 50.0%); for many of these respondents, the most recent type of disability leave was long-term (MF, 57.6%; PV, 43.6%; ET, 34.6%). For respondents who returned to employment after their most recent medical disability leave (MF, 22.8%; PV, 49.1%; ET, 50.0%), the average lengths of leave were 5.7, 4.6, and 2.2 months, respectively.

The average time from diagnosis to the first employment change for respondents who transitioned from full- to part-time employment was 1.4 years (MF), 5.4 years (PV), and 3.5 years (ET). For those who reported reducing work hours for at least 3 months, the average time from diagnosis to the first reduction in work hours was 1.0 year (MF), 4.0 years (PV), and 2.6 years (ET).

### Impact of Myeloproliferative neoplasms on career potential

**Fig. 2:**
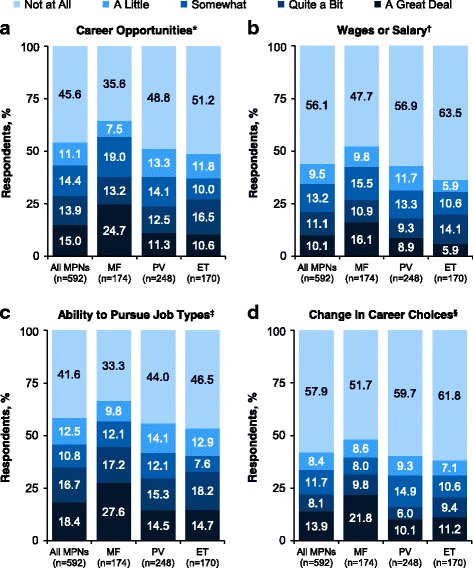
Impact of MPNs on career potential among respondents employed at MPN diagnosis. Measures of career potential were **(a)** career opportunities, **(b)** wages or salary, **(c)** ability to pursue job types, and **(d)** change in career choices. ET, essential thrombocythemia; MF, myelofibrosis; MPN, myeloproliferative neoplasm; PV, polycythemia vera. *As a result of your MF/PV/ET, have you ever been limited in your career opportunities? ^†^As a result of your MF/PV/ET, have you ever been limited in your wages/salary (from employment, investment, etc)? ^‡^As a result of your MF/PV/ET, have you ever been limited in your ability to pursue certain types of jobs or careers? ^§^As a result of your MF/PV/ET, have you ever been forced to change your career choices?

The effects of an MPN diagnosis on career potential were assessed among respondents who were employed at diagnosis using 4 questions (Figure 2). More than half of the respondents reported that their career opportunities had been limited as a result of their MPN (54.4%); wages or salaries were limited for 43.9% of respondents. A majority of respondents indicated that they had a limited ability to pursue certain types of jobs (58.4%), and 42.1% of respondents reported that their MPN diagnosis forced a change in career choices. Among respondents reporting limitations, the highest grade of impact (ie, “a great deal”) was reported by 23.1%–32.9% across all 4 questions.

### Impact of Myeloproliferative neoplasms on work productivity

At the time of survey participation, 398 respondents were employed full- or part-time (MF, *n* = 86; PV, *n* = 177; ET, *n* = 135). Among these respondents, 40.7% (MF), 29.4% (PV), and 31.1% (ET) reported missing work in the past 7 days. The average reported amount of work time missed in the past 7 days was 6.2, 7.2, and 6.7 h, respectively (Table 3).

**Table 3:** Employment Status and Missed Work Time Among Respondents Employed at Time of Survey Participation

	MF (*n* = 86)	PV (*n* = 177)	ET (*n* = 135)	All MPNs (*n* = 398)
Current employment status, n (%)				
Full-time (≥40 h/wk)	53 (61.6)	127 (71.8)	108 (80.0)	288 (72.4)
Part-time (< 40 h/wk)	33 (38.4)	50 (28.2)	27 (20.0)	110 (27.6)
Respondents who reported missing work, n (%)	35 (40.7)	52 (29.4)	42 (31.1)	129 (32.4)
Mean (SD) missed work time in the past 7 d because of disease,* h	6.2 (5.6)	7.2 (9.0)	6.7 (5.5)	6.8 (7.1)

ET, essential thrombocythemia; MF, myelofibrosis; MPN, myeloproliferative neoplasm; PV, polycythemia vera

*Mean work time missed is reported only for respondents who reported missing work

**Fig. 3:**
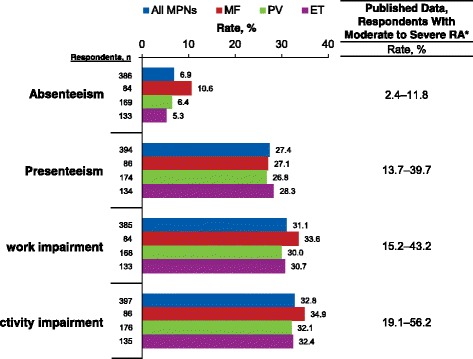
WPAI-SHP scores among currently employed respondents. ET, essential thrombocythemia; MF, myelofibrosis; MPN, myeloproliferative neoplasm; PV, polycythemia vera; RA, rheumatoid arthritis; WPAI-SHP, Work Productivity and Activity Impairment Specific Health Problem questionnaire. All items based on 7-day recall (ie, regarding work/activity during the 7 days preceding the survey). *Ranges for published data include scores at baseline and after active treatment for RA in studies of European patients with moderate RA (Pavelka et al. 2013 [15]) and patients from the United States with moderate to severe RA (Hone et al. 2013 [13])

Overall, mean WPAI-SHP scores for absenteeism and presenteeism within the previous 7-day period were 6.9% and 27.4%, respectively (Figure 3). Mean scores for overall work impairment or activity impairment within the previous 7-day period were 31.1% and 32.8%, respectively (Figure 3).

**Fig. 4:**
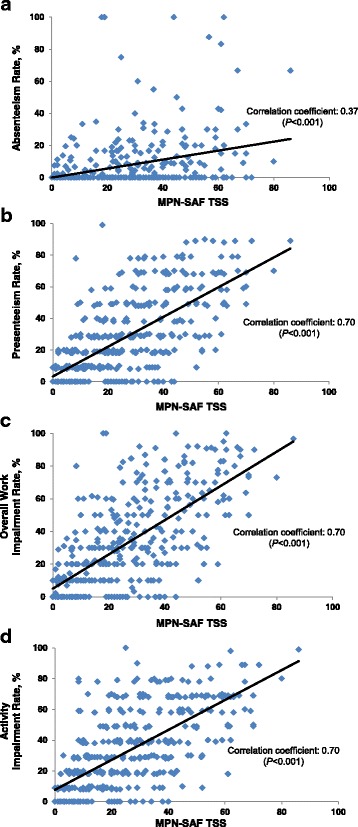
Correlations between MPN-SAF TSS and WPAI-SHP scores among currently employed respondents. MPN-SAF TSS were correlated to WPAI-SHP scores for **(a)** absenteeism (*n* = 386), **(b)** presenteeism (*n* = 394), **(c)** overall work productivity (*n* = 385), and **(d)** activity impairment (*n* = 397). ET, essential thrombocythemia; MF, myelofibrosis; MPN, myeloproliferative neoplasm; MPN-SAF TSS, Myeloproliferative Neoplasm Symptom Assessment Form Total Symptom Score; PV, polycythemia vera; WPAI-SHP, Work Productivity and Activity Impairment Specific Health Problem questionnaire

The mean MPN-SAF TSS score was 24.8 for all respondents, and 25.1, 25.5 and 23.9, respectively, for respondents with MF, PV, and ET. MPN-SAF TSS and most of the WPAI-SHP scores were highly correlated, and the correlation coefficients were as follows for all respondents: absenteeism, 0.37; presenteeism, 0.70; work impairment, 0.70; activity impairment, 0.70 (all *P* < 0.001; Figure 4). A significant correlation was observed for each of the 10 symptoms in the MPN-SAF TSS, with the highest correlation coefficients occurring for the following symptoms: problems with concentration, inactivity, and fatigue (Additional file 2: Table S2). The degree of correlation for each symptom was consistent across the 3 MPN diseases.

## Discussion

This analysis of the Living With MPNs survey indicated that MPNs can have a substantial negative impact on patients’ employment, career potential, and work productivity. Approximately half of employed respondents experienced at least one change in employment following diagnosis because of their disease. The most frequent changes were “left a job” (30.2%), “went on medical disability leave” (24.8%), and “had reductions in work hours for at least 3 months” (21.8%). The first change in employment status occurred approximately 2 years after the MPN diagnosis.

The adverse impacts of MPNs on employment were as severe as the effects observed in some patients with other terminal cancers or chronic debilitating/disabling diseases that have a significant symptom burden. In a study of patients with metastatic breast, colorectal, lung, or prostate cancer, 58% reported an unstable work status, including 45% who had stopped working because of their disease [12]. In a study of patients with moderate to severe rheumatoid arthritis, 17% reported changing jobs because of their disease [13]. In another study, 61.2% with heart disease and 44.5% with arthritis stopped working because of their disease [14].

The degree of work productivity impairment caused by MPNs was also comparable to reports by patients with other chronic disorders [13, 15–18]. Patients with moderate to severe rheumatoid arthritis reported impairments on work productivity and activity (range of mean scores: absenteeism, 2.4–11.8; presenteeism, 13.7–39.7; overall work impairment, 15.2–43.2; activity impairment, 19.1–56.2) [13, 15] that were similar to those reported by respondents in the Living With MPNs survey (Figure 3). Furthermore, the proportion of respondents reporting “taking medical disability leave” in the survey (24.8%) paralleled previous results of patients who received a work disability pension because of rheumatoid arthritis (19%) [19]. Similar impairments to work productivity have also been reported by patients with chronic bronchitis, emphysema, chronic obstructive pulmonary disease [16], severe asthma [17], and painful diabetic neuropathy [18].

Chronic diseases are known to place significant financial burdens on patients in the workforce [8, 20]. In an analysis of 1117 adults (25–64 years of age) diagnosed with any type of cancer or malignant tumor interviewed between 1999 and 2009, notable reductions in employment status, total work hours, earnings, and total family income were found during the 5 years after cancer diagnosis [8]. Based on the impact of MPNs on employment status, career opportunities, and quality of life, there may be potentially considerable financial losses suffered by the respondents, which can perhaps extend to their immediate family members; this financial impact may be examined in a future analysis. Negative changes in patients’ work life can also have important non-financial implications. In other cancer settings, such changes have been linked to a diminished quality of life [12], psychiatric disorders (eg, depression) [13], and feelings of a suspension of life and/or normalcy [12, 14].

Analyses of symptom data using the MPN-SAF TSS suggest that greater symptom burden, which may be indicative of a more advanced stage and/or suboptimal disease, was associated with greater impairment in work productivity and daily activity among patients with MPNs. The most prevalent symptoms among patients with MPNs (eg, fatigue, abdominal discomfort, night sweat, itching, and concentration problems) were each associated with impairment in work productivity and daily activity. Effectively managing patients with MPNs by ameliorating their symptoms may help improve their work productivity and their ability to participate in the daily activities of life. Other supportive interventions may also help improve patients’ work life, including local and national services for patients with chronic diseases, professional caregiving, transportation alternatives for traveling to and from work, and opportunities that may be available through the Family and Medical Leave Act [21].

There are a few limitations of this patient self-reported study. First, there may have been a selection bias regarding who completed the survey; the educational levels of respondents were higher compared with the general US population, per 2015 US Census data [22]. Second, recruitment methods were limited to patients reachable through targeted emails and postcard mailings, social media and select websites, select hematologist/oncologist offices, and Google search advertising. These factors may have limited the ability to generalize the findings to all patients, but similar limitations were reported in previous online surveys of patients with MPNs [3, 23], and the symptom burden of patients in the current survey was comparable to that of patients with MPNs in an international survey administered at physician offices [2]. Third, because patient-reported disease stage/severity may not be accurate, limited MPN severity data were collected by the survey, which precluded an ability to assess how employment was impacted by different disease stages. Finally, the analysis excluded respondents who were self-employed to focus on patients in more structured work environments.

## Conclusions

All 3 types of MPNs have a substantial negative impact on patients’ employment status, career potential, and work productivity comparable to the impairment levels reported for other disabling chronic conditions. MF is sometimes thought of as the most severe MPN. However, a notable proportion of patients with PV and ET are also severely impacted by the symptoms and corresponding impairments associated with their disease. Approximately 50% of employed patients with MPNs had a change in employment status because of their disease. The higher the symptom burden, the greater the detrimental impact on work productivity and daily activity. Effective and timely management of MPNs and related symptoms could reduce the adverse impact on employment and work productivity and potentially abate losses in income.

## Additional files

Additional file 1: **Table S1.** Survey Questions Reported in This Analysis. (DOCX 55 kb)

Additional file 2: **Table S2.** Correlation Between WPAI-SHP Scores and MPN-SAF Symptom Scores. (DOCX 54 kb)

## Abbreviations

COPD: Chronic obstructive pulmonary disease
ET: Essential thrombocytopenia
MF: Myelofibrosis
MPN: Myeloproliferative neoplasm
MPN-SAF TSS: Myeloproliferative Neoplasm Symptom Assessment Form Total Symptom Score
PV: Polycythemia vera
RA: Rheumatoid arthritis
WPAI-SHP: Work Productivity and Activity Impairment Specific Health Problem questionnaire
