# Supplementary material for: Impact of Myeloproliferative neoplasms on patients’ employment status and work productivity in the United States: results from the living with MPNs survey
Source: BMC Cancer. 2018 Apr 13;18:420. doi: 10.1186/s12885-018-4322-9 (PMC5899342; doi:10.1186/s12885-018-4322-9)
Supplement: Supplementary file 1 — Table S1. Survey Questions Reported in This Analysis. (DOCX 55 kb) [file 12885_2018_4322_MOESM1_ESM.docx]

Impact of Myeloproliferative Neoplasms on Patients’ Employment Status and Work Productivity in the United States: Results From the Living With MPNs Survey

Jingbo Yu, MHA, PhD,^1^ Shreekant Parasuraman, BPharm, PhD,^1^ Dilan Paranagama, PhD,^1^ Andrew Bai, MS,^1^ Ahmad Naim, MD,^1^ David Dubinski, MBA,^1^ Ruben Mesa, MD, FACP^2^

^1^Incyte Corporation, 1801 Augustine Cut-Off, Wilmington, DE, 19803; ^2^Mayo Clinic, 13400 E. Shea Blvd, Scottsdale, AZ, 85259

## SUPPLEMENTAL MATERIALS

## Supplemental Table 1. Survey Questions Reported in This Analysis

| Number | Question | Condition |
| --- | --- | --- |
| **Employment Status** | |  |
| Q1 | As a result of your MF/PV/ET, have you ever left a job? | All respondents |
| Q2 | How many times have you left a job as a result of your MF/PV/ET? | Yes on Q1 |
| Q3 | Please enter the year(s) for the first, second, and most recent times you left a job as a result of your MF/PV/ET | Yes on Q1 |
| Q4 | About the most recent time when you left a job due to your MF/PV/ET: How much was your annual salary/wage at the time you left your previous job? | Yes on Q1 |
| Q5 | About the most recent time when you left a job due to your MF/PV/ET: After you left, did you find another permanent job? | Yes on Q1 |
| Q6 | About the most recent time when you left a job due to your MF/PV/ET: How much was your annual salary/wage at your new job? | Yes on Q5 |
| Q7 | As a result of your MF/PV/ET, have you ever taken early retirement? | All employed respondents |
| Q8 | When did you take early retirement? | Yes on Q7 |
| Q9 | How many years in advance did you retire compared to what you had originally planned? | Yes on Q7 |
| Q10 | As a result of your MF/PV/ET, have you ever gone on medical disability leave (including short term disability, long term disability, or other disability)? | All employed respondents |
| Q11 | Please enter the year(s) for the first, second, and most recent time that you went on medical disability leave as a result of your MF/PV/ET. | Yes on Q10 |
| Q12 | About the most recent time you went on medical disability due to your MF/PV/ET: Did you take the leave as short-term or long-term disability leave? | Yes on Q10 |
| Q13 | About the most recent time you went on medical disability due to your MF/PV/ET: After you left on disability, did you return to that job? | Yes on Q10 |
| Q14 | About the most recent time you went on medical disability due to your MF/PV/ET: How long did it take you to return to that job? | Yes on Q13 |
| Q15 | Have you ever applied for Social Security disability benefit due to your MF/PV/ET? | All respondents |
| Q16 | Have you ever received Social Security disability benefit due to your MF/PV/ET? | Yes on Q15 |
| Q17 | As a result of your MF/PV/ET, have you ever changed from full-time employment to part-time employment? | All employed respondents |
| Q18 | How many times have you ever changed from full-time to part-time employment a result of your MF/PV/ET? | Yes on Q17 |
| Q19 | Please enter the year(s) for the first, second, and most recent times you changed from full-time to part-time employment as a result of your MF/PV/ET | Yes on Q15 |
| Q20 | About the most recent time you changed from full-time to part-time employment due to your MF/PV/ET: Did you ever return to full-time, whether at that job or another job? | Yes on Q17 |
| Q21 | About the most recent time you changed from full-time to part-time employment due to your MF/PV/ET: How long did it take you to return to full-time, whether at that job or another job? | Yes on Q20 |
| Q22 | As a result of your MF/PV/ET, have you ever had any other reductions in your hours at work for 3 months or more? | All employed respondents |
| Q23 | Please enter the year(s) for the first, second, and most recent times that you had other reductions in your hours at work for 3 months or more as a result of your MF/PV/ET | Yes on Q17 |
| Q24 | About the most recent time when you reduced work hours due to your MF/PV/ET: How many hours a week (on average) did you work before and after your hours were reduced? | Yes on Q22 |
| Q25 | About the most recent time when you reduced work hours due to your MF/PV/ET: Did you ever return to your previous work week hours, either at that job or another job? | Yes on Q22 |
| Q26 | About the most recent time when you reduced work hours due to your MF/PV/ET: How long did it take you to return to your previous work week hours, either at that job or another job? | Yes on Q25 |
| Q27 | As a result of your MF/PV/ET, were you ever reassigned to or did you take another job at a lower salary or wage? | All employed respondents |
| **Career Potential** | |  |
| Q28 | As a result of your MF/PV/ET have you ever been limited in your career opportunities? | All respondents |
| Q29 | As a result of your MF/PV/ET have you ever been limited in your wages/salary (from employment, investment, etc.)? | All respondents |
| Q30 | As a result of your MF/PV/ET have you ever been limited in your ability to pursue certain types of jobs or careers | All respondents |
| Q31 | As a result of your MF/PV/ET have you ever been forced to change your career choices? | All respondents |
| **Work Productivity (WPAI-SHP)** | |  |
| Q32 | During the past 7 days, how many hours did you miss from work because of problems associated with your MF/PV/ET? Include hours you missed on sick days, medical appointment, times you went in late, left early, etc., because of your MPN. Do not include time you missed to participate in this study. | All employed respondents |
| Q33 | During the past 7 days, how many hours did you actually work? | All employed respondents |
| Q34 | During the past 7 days, how much did your MF/PV/ET affect your productivity while you were working? On a scale from 0 (no effect) to 10 (prevented me from working) | All employed respondents |
| Q35 | During the past 7 days, how much did your MF/PV/ET affect your ability to do your regular daily activities, other than work at a job? On a scale from 0 (no effect) to 10 (prevented me from doing my daily activities) | All respondents |

ET, essential thrombocytopenia; MF, myelofibrosis; PV, polycythemia vera; WPAI-SHP, Work Productivity and Activity Impairment Specific Health Problem questionnaire.
