# Supplementary material for: Impact of Myeloproliferative neoplasms on patients’ employment status and work productivity in the United States: results from the living with MPNs survey
Source: BMC Cancer. 2018 Apr 13;18:420. doi: 10.1186/s12885-018-4322-9 (PMC5899342; doi:10.1186/s12885-018-4322-9)
Supplement: Supplementary file 2 — Table S2. Correlation Between WPAI-SHP Scores and MPN-SAF Symptom Scores. (DOCX 54 kb) [file 12885_2018_4322_MOESM2_ESM.docx]

Impact of Myeloproliferative Neoplasms on Patients’ Employment Status and Work Productivity in the United States: Results From the Living With MPNs Survey

Jingbo Yu, MHA, PhD,^1^ Shreekant Parasuraman, BPharm, PhD,^1^ Dilan Paranagama, PhD,^1^ Andrew Bai, MS,^1^ Ahmad Naim, MD,^1^ David Dubinski, MBA,^1^ Ruben Mesa, MD, FACP^2^

^1^Incyte Corporation, 1801 Augustine Cut-Off, Wilmington, DE, 19803; ^2^Mayo Clinic, 13400 E. Shea Blvd, Scottsdale, AZ, 85259

## SUPPLEMENTAL MATERIALS

## Supplemental Table 2. Correlation Between WPAI-SHP Scores and MPN-SAF Symptom Scores.

| **WPAI-SHP Component** | **MPN-SAF Symptom** | **Spearman's Correlation Coefficient*** |
| --- | --- | --- |
| Absenteeism | MPN-SAF TSS (n=386) | 0.37 |
|  | Problems with concentration (n=386) | 0.34 |
|  | Inactivity (n=386) | 0.32 |
|  | Fever (n=306) | 0.31 |
|  | Fatigue (n=306) | 0.29 |
|  | Unintentional weight loss (n=386) | 0.26 |
|  | Bone pain (n=306) | 0.26 |
|  | Early satiety (n=386) | 0.24 |
|  | Night sweats (n=386) | 0.22 |
|  | Itching (n=386) | 0.22 |
|  | Abdominal discomfort (n=306) | 0.21 |
| Presenteeism | MPN-SAF TSS (n=394) | 0.70 |
|  | Problems with concentration (n=394) | 0.68 |
|  | Inactivity (n=394) | 0.63 |
|  | Fatigue (n=313) | 0.59 |
|  | Bone pain (n=313) | 0.50 |
|  | Early satiety (n=394) | 0.45 |
|  | Itching (n=394) | 0.45 |
|  | Abdominal discomfort (n=313) | 0.44 |
|  | Fever (n=313) | 0.42 |
|  | Night sweats (n=394) | 0.42 |
|  | Unintentional weight loss (n=394) | 0.37 |
| Work impairment | MPN-SAF TSS (n=385) | 0.70 |
|  | Problems with concentration (n=385) | 0.67 |
|  | Inactivity (n=385) | 0.62 |
|  | Fatigue (n=305) | 0.57 |
|  | Bone pain (n=305) | 0.49 |
|  | Early satiety (n=385) | 0.46 |
|  | Itching (n=385) | 0.44 |
|  | Abdominal discomfort (n=305) | 0.43 |
|  | Fever (n=305) | 0.43 |
|  | Night sweats (n=385) | 0.42 |
|  | Unintentional weight loss (n=385) | 0.39 |
| Activity impairment | MPN-SAF TSS (n=397) | 0.70 |
|  | Inactivity (n=397) | 0.71 |
|  | Problems with concentration (n=397) | 0.64 |
|  | Fatigue (n=316) | 0.62 |
|  | Bone pain (n=316) | 0.47 |
|  | Early satiety (n=397) | 0.44 |
|  | Abdominal discomfort (n=316) | 0.43 |
|  | Itching (n=397) | 0.42 |
|  | Night sweats (n=397) | 0.41 |
|  | Fever (n=316) | 0.37 |
|  | Unintentional weight loss (n=397) | 0.30 |

MPN-SAF TSS, Myeloproliferative Neoplasm Symptom Assessment Form Total Symptom Score; WPAI-SHP, Work Productivity and Activity Impairment Specific Health Problem questionnaire.

**P*<0.001 for all correlations.
